# The Extent and Consequences of P-Hacking in Science

**DOI:** 10.1371/journal.pbio.1002106

**Published:** 2015-03-13

**Authors:** Megan L. Head, Luke Holman, Rob Lanfear, Andrew T. Kahn, Michael D. Jennions

**Affiliations:** 1 Division of Evolution, Ecology and Genetics, Research School of Biology, Australian National University, Acton, Canberra, Australia; 2 Department of Biological Sciences, Faculty of Science, Macquarie University, North Ryde, New South Wales, Australia

## Abstract

A focus on novel, confirmatory, and statistically significant results leads to substantial bias in the scientific literature. One type of bias, known as “p-hacking,” occurs when researchers collect or select data or statistical analyses until nonsignificant results become significant. Here, we use text-mining to demonstrate that p-hacking is widespread throughout science. We then illustrate how one can test for p-hacking when performing a meta-analysis and show that, while p-hacking is probably common, its effect seems to be weak relative to the real effect sizes being measured. This result suggests that p-hacking probably does not drastically alter scientific consensuses drawn from meta-analyses.

## Abstract 2

Publication bias resulting from so-called "p-hacking" is pervasive throughout the life sciences; however, its effects on general conclusions made from the literature appear to be weak.

## Introduction

There is increasing concern that many published results are false positives [1,2] (but see [3]). Many argue that current scientific practices create strong incentives to publish statistically significant (i.e., “positive”) results, and there is good evidence that journals, especially prestigious ones with higher impact factors, disproportionately publish statistically significant results [4–10]. Employers and funders often count papers and weigh them by the journal’s impact factor to assess a researcher’s performance [11]. In combination, these factors create incentives for researchers to selectively pursue and selectively attempt to publish statistically significant research findings.

There are two widely recognized types of researcher-driven publication bias: selection (also known as the “file drawer effect”, where studies with nonsignificant results have lower publication rates [7]) and inflation [12]. Inflation bias, also known as “p-hacking” or “selective reporting,” is the misreporting of true effect sizes in published studies (Box 1). It occurs when researchers try out several statistical analyses and/or data eligibility specifications and then selectively report those that produce significant results [12–15]. Common practices that lead to p-hacking include: conducting analyses midway through experiments to decide whether to continue collecting data [15,16]; recording many response variables and deciding which to report postanalysis [16,17], deciding whether to include or drop outliers postanalyses [16], excluding, combining, or splitting treatment groups postanalysis [2], including or excluding covariates postanalysis [14], and stopping data exploration if an analysis yields a significant *p*-value [18,19].

If published data are biased, data synthesis might lead to flawed conclusions. Meta-analysis is a set of statistical methods that combine studies on the same question to estimate the true effect size [33]. Meta-analyses are now the “gold standard” for synthesizing the evidence for an effect of a treatment or the existence of a relationship, and combining effect size estimates across studies to give an overall estimate. Meta-analyses guide the application of medical treatments and policy decisions, and influence future research directions [34]. However, meta-analyses are compromised if the studies being synthesized do not reflect the true distribution of effect sizes [5,35–37].

Quantifying p-hacking is important because publication of false positives hinders scientific progress. When false positive results enter the literature they can be very persistent. In many fields, there is little incentive to replicate research [38]. Even when research is replicated, early positive studies often receive more attention than later negative ones. In addition, false positives can inspire investment in fruitless research programs, and even discredit entire fields [14,16].

Despite the potential importance of p-hacking, the consequences for formal and informal data synthesis are unknown. Here, we address both issues using p-curves (see Box 2). First, we used text-mining to obtain reported p-values in papers drawn from a broad range of scientific disciplines. We then looked for evidence of p-hacking based on the shape of the p-curves. Second, we produced p-curves from primary data used in published meta-analyses. This allowed us to test the evidence for p-hacking when looking at specific hypotheses which researchers have clearly identified as being of general interest (i.e., that warrant a meta-analysis).

**Box 1. The History of P**

Fisher [20] introduced null hypothesis significance testing (NHST) to objectively separate interesting findings from background noise [21]. NHST is the most widely used data analysis method in most scientific disciplines [22,23]. The null hypothesis is typically a statement of no relationship between variables or no effect of an experimental manipulation. With NHST, one computes the probability (i.e., p) of finding an effect at least or more extreme than the observed finding if the null hypothesis is true [24,25].

The NHST approach uses an arbitrary cutoff value (usually 0.05). Findings with smaller *p*-values are described as “statistically significant” (“positive” findings), and the remainder as “nonsignificant” (“negative” findings). This arbitrary cutoff has led to the scientifically dubious practice of regarding “significant” findings as more valuable, reliable, and reproducible [24], thereby incentivizing various kinds of research bias.

Before computers, test statistics (e.g., *t* and *F*) were routinely calculated by hand and the associated *p*-value was looked up in statistical tables. Here, *p*-values were given for a limited set of values (e.g., 0.001, 0.01, 0.02, and 0.05) [26]. Researchers then reported *p*-values as the lowest threshold consistent with the test statistic (e.g., *p* < 0.05 or *p* < 0.01). With modern statistical software this practice is unnecessary, as precise *p*-values are now provided, but it is still commonplace. Previous research has shown that strict adherence to *p*-value thresholds can bias how research is reported, even within the region of significance [27].

The *p*-value is easily misinterpreted. For example, it is often equated with the strength of a relationship, but a tiny effect size can have very low *p*-values with a large enough sample size. Similarly, a low *p*-value does not mean that a finding is of major clinical or biological interest [28]. Many researchers have advocated abolishing NHST (e.g., [29,30]). However, others note that many of the problems with publication bias reoccur with other approaches, such as reporting effect sizes and their confidence intervals [31] or Bayesian credible intervals [32]. Publication biases are not a problem with *p*-values per se. They simply reflect the incentives to report strong (i.e., significant) effects.

**Box 2. The P-Curve: What Can It Tell Us?**

A p-curve is the distribution of *p*-values for a set of studies. P-curves can be a helpful tool to assess the reliability of published research. Here, we outline how they have been used to assess the literature.

**Evidential value**

One can examine whether a set of findings contains evidential value by examining the distribution of *p*-values, particularly those between 0 and 0.05. “Evidential value” refers to whether or not the published evidence for a specific hypothesis suggests that the effect size is nonzero.

**Fig 1:**
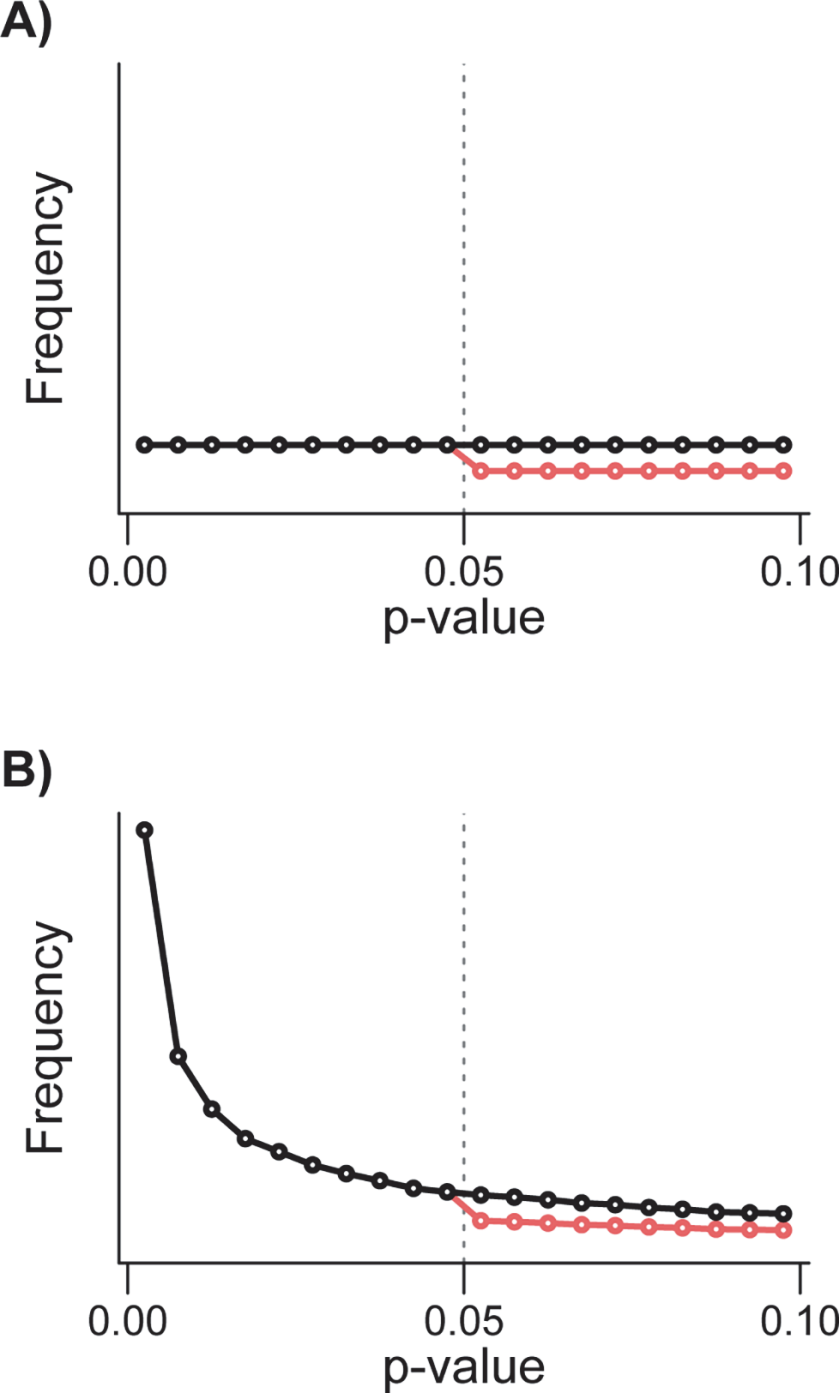
The effect of publication bias on the distribution of *p*-values around the significance threshold of 0.05. A) Black line shows distribution of p-values when there is no evidential value and the red line shows how publication bias influences this distribution. B) Black line shows distribution of *p*-values when there is evidential value and red line shows how publication bias influences this distribution. Tests for publication bias due to a file-drawer effect often compare the number of *p*-values in each of the bins either side of 0.05.

**Fig 2:**
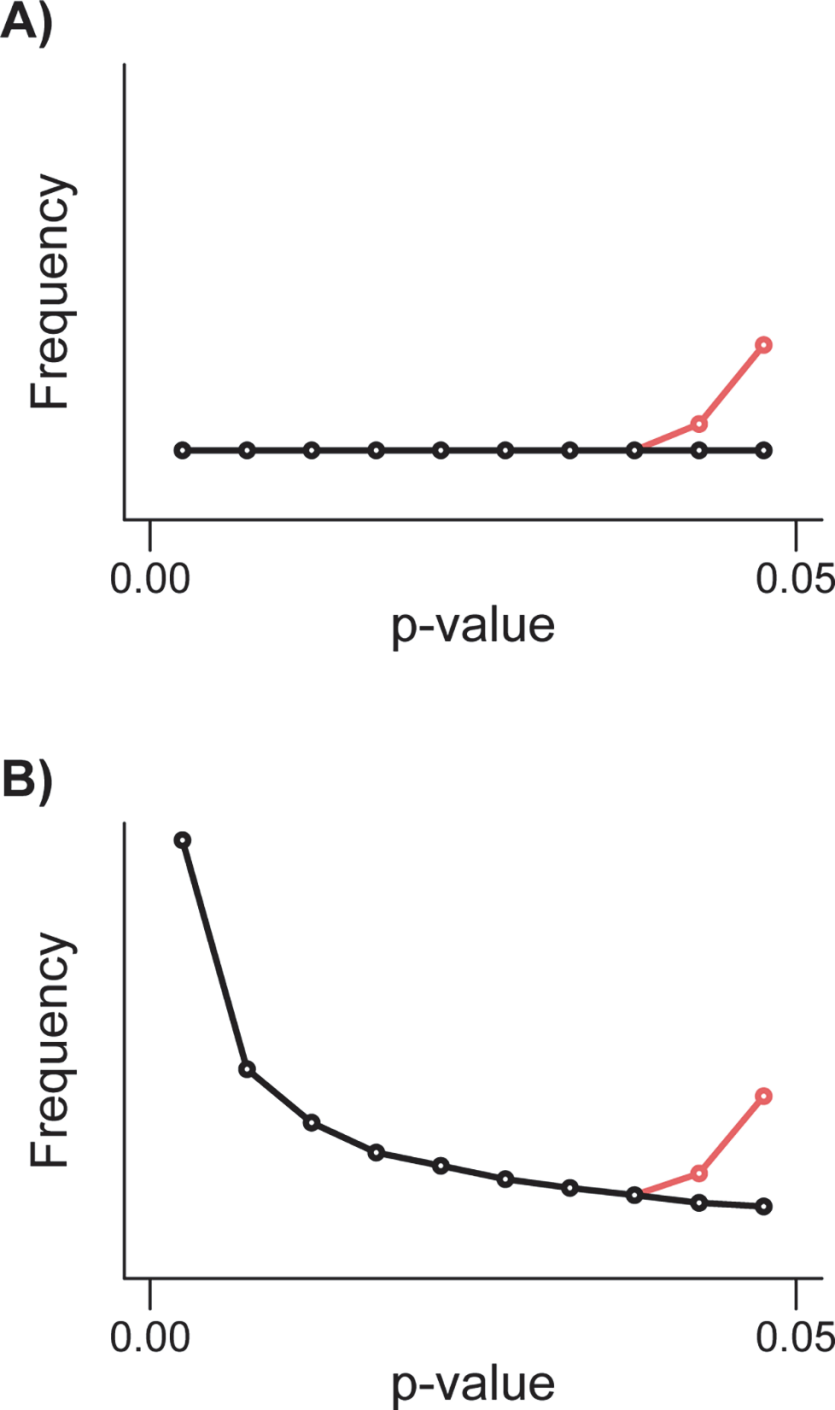
The effect of p-hacking on the distribution of p-values in the range of significance. A) Black line shows distribution of *p*-values when there is no evidential value and the red line shows how p-hacking influences this distribution. B) Black line shows distribution of *p*-values when there is evidential value and the red line shows how p-hacking influences this distribution. Tests for p-hacking often compare the number of *p*-values in two adjacent bins just below 0.05.

When the effect size for a studied phenomenon is zero, every *p*-value is equally likely to be observed. The expected distribution of *p*-values under the null hypothesis is uniform (Black line, Fig. 1A and Fig. 2A), such that *p*<0.05 will occur 5% of the time, *p*<0.04 will occur 4% of the time, and so on. On the other hand, when the true effect size is nonzero, the expected distribution of *p*-values is exponential with a right skew [39–42] (Black line, Fig. 1B and Fig. 2B). When the true effect is strong, researchers are more likely to obtain very low *p*-values (e.g., *p*<0.001) than moderately low *p*-values (e.g., 0.01), and less likely still to obtain nonsignificant *p*-values (p > 0.05) [41]. So, as the true effect size increases the p-curve is more right skewed [41].

**Publication bias**

Several studies have plotted the distribution of *p*-values or related test statistics (i.e., *Z* or *t*) around the main significance threshold of *p* = 0.05 (often in the range of 0.01 to 0.1). A notable drop in *p*-values above 0.05 (or for Z values, 1.96) (Red line, Fig. 1A and Fig. 1B) is interpreted as evidence for publication bias (e.g., [40,43–45]). While a discontinuity in the distribution of *p*-values around 0.05 is indicative of publication bias, it does not distinguish between selective publication bias and p-hacking (see Box 1).

**P-hacking**

The p-curve can, however, be used to identify p-hacking, by only considering significant findings [14]. If researchers p-hack and turn a truly nonsignificant result into a significant one, then the p-curve’s shape will be altered close to the perceived significance threshold (typically *p* = 0.05). Consequently, a p-hacked p-curve will have an overabundance of *p*-values just below 0.05 [12,40,41]. If researchers p-hack when there is no true effect, the p-curve will shift from being flat to left skewed (Fig. 2A). If, however, researchers p-hack when there is a true effect, the p-curve will be exponential with right skew but there will be an overrepresentation of *p*-values in the tail of the distribution just below 0.05 (Fig. 2B). Both p-hacking and selective publication bias predict a discontinuity in the p-curve around 0.05, but only p-hacking predicts an overabundance of *p*-values just below 0.05 [12]. The exact shape of the p-curve will, however, depend on both the true effect (i.e., the p-curve before p-hacking) and the intensity of p-hacking [41].

**Assessing p-curves for p-hacking and evidential value**

Similar to previous studies (e.g., [14,43]) we employ binomial tests to look for evidence of evidential value and p-hacking in both our text-mined and meta-analyses datasets. We tested for evidential value using a two-tailed sign test, in which we compared the number of *p*-values falling in the bin 0 ≤ *p* < 0.025 to the number in the bin 0.025 ≤ *p* < 0.05. Under the null hypothesis of no evidential value, the expected number of *p*-values in each of these bins is equal. Significantly more *p*-values in the lower bin is consistent with evidential value (i.e., right skewed p-curve), and significantly more *p*-values in the upper bin is consistent with severe p-hacking. This test is a slightly modified version of a test proposed by Simohnson et al. [41], who suggest using two separate one-tailed sign tests for the same purpose.

The two-tailed sign test with a *p* = 0.025 threshold (above) and the tests proposed by Simonsohn et al. [41] can detect severe p-hacking, but are insensitive to more modest (and arguably more realistic) levels of p-hacking. This is true especially if the average true effect size is strong, as the right skew introduced to the p-curve will mask the left skew caused by p-hacking. A more sensitive approach to detect p-hacking is to look for an increase in the relative frequency of *p*-values just below 0.05, where we expect the signal of p-hacking to be strongest. Under the null hypothesis of no p-hacking, we expect either that the distribution of *p*-values is uniform close to 0.05 (if the true effect sizes are zero), or right skewed (i.e., if at least some effect sizes are nonzero). However, p-hacking introduces additional *p*-values close to 0.05, producing a left skew. Thus, a simple, and conservative, test for p-hacking involves testing the null hypothesis that the *p*-values just below 0.05 are either uniformly distributed or right skewed. We used a one-tailed sign test to ask whether the number of *p*-values in the bin that abuts 0.05 is greater than that in the adjacent lower bin. This test becomes more likely to detect p-hacking if one uses smaller bins, since *p*-values are right skewed when the average effect size is positive (masking p-hacking), but in practice, using smaller bins will reduce the sample size (and thus power) of the test. We selected a bin width of 0.005, with the lower bin specified as 0.04 < *p* < 0.045 and the upper bin as 0.045 < p < 0.05. We chose *p* < 0.05 as the cutoff for our upper bin (following [3]), rather than *p* = 0.05 (see [46]) because we suspect that many authors do not regard *p* = 0.05 as significant. As a measure of the strength of p-hacking, we present the proportion of *p*-values in the upper bin and the associated 95% confidence intervals (calculated following Clopper and Pearson [47] using the *binom.test* function in R).

We ran the above analyses separately for each discipline and meta-analysis dataset. In addition, we tested for overall evidential value (two-tailed test) and signs of p-hacking (one-tailed test) in the two main datasets (Text-mining of *p*-values and the meta-analysis data sets respectively). To do this, we used the proportion of *p*-values occurring in the upper bin for each discipline or meta-analysis (depending on the dataset being analysed) and ran a binomial generalised linear model to test whether the observed intercept differed from 0.5 (i.e., equal number of cases in the two bins). This approach is equivalent to a meta-analysis testing for a significant trend when combining the individual disciplines or questions because each is weighted by its sample size. The R code we used is deposited in Dryad [48].

## Assessing the Extent of P-Hacking in the Scientific Literature Using Text-Mining

We used text-mining to search for *p*-values in all Open Access papers available in the PubMed database (see S1 Text). To quantify “evidential value” (i.e., if there is evidence that the true effect size is nonzero) and p-hacking, we constructed p-curves from the *p*-values we obtained (see Box 2). We present separate tests of evidential value and p-hacking for *p*-values extracted from the Results section, and for *p*-values extracted from the Abstracts. Researchers have identified weaknesses in the use of text-mined data to look for publication bias (e.g., [46]). Here, we adopted several measures to counter these weaknesses (see S1 Text).

**Fig 3:**
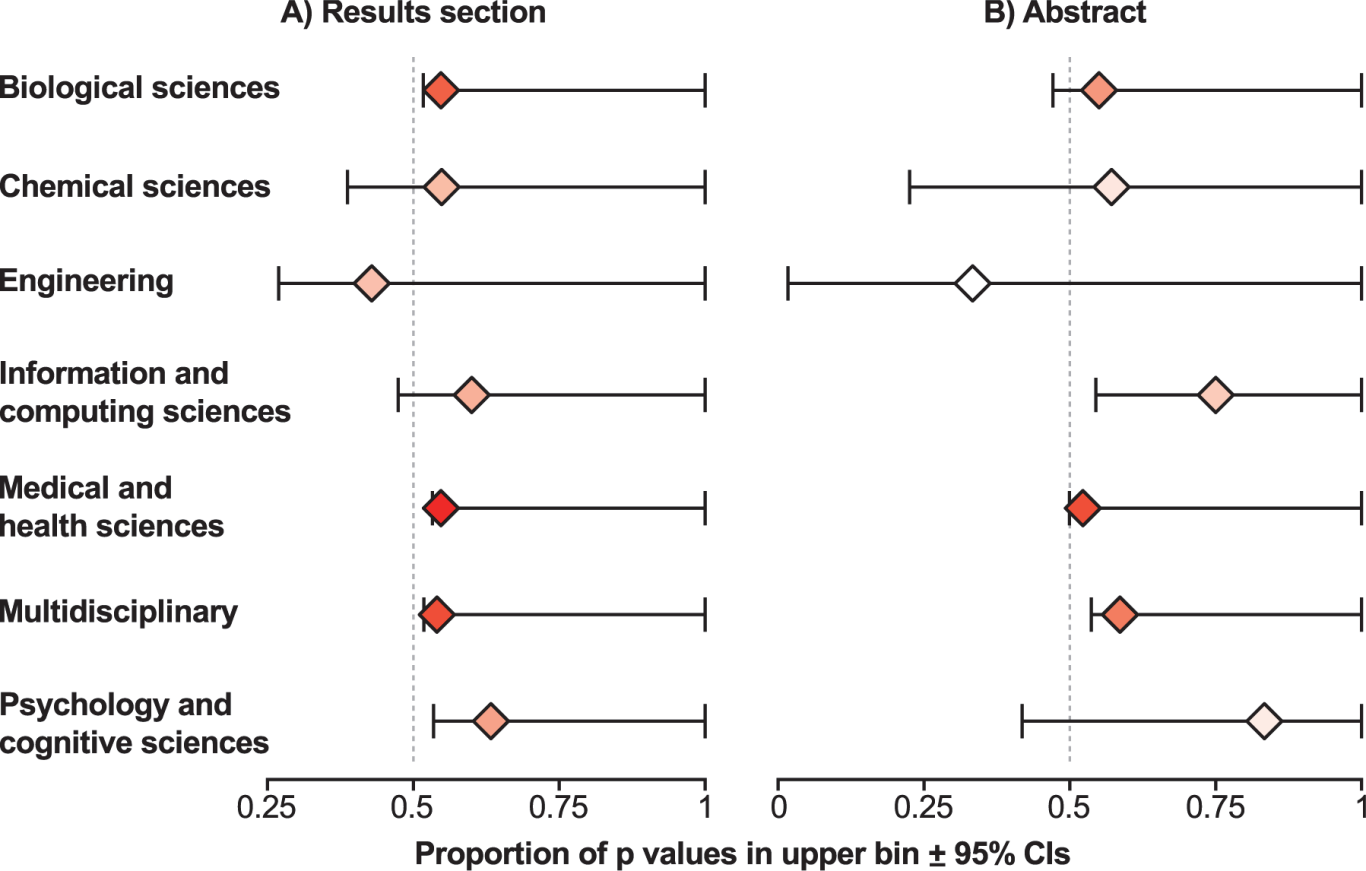
Evidence for p-hacking across scientific disciplines. A) Evidence for p-hacking from *p*-values obtained from Results sections. B) Evidence for p-hacking from *p*-values obtained from Abstracts. The strength of p-hacking is presented as the proportion of p-values in the upper bin (0.045 < p < 0.05) with one-tailed 95% confidence intervals (calculated following Clopper and Pearson [47] using the *binom.test* function in R). Only disciplines where text-mining of the Results sections returned more than 25 *p*-values between 0.04 and 0.05 are presented. Marker colour is shaded according to the sample size: with white indicating low samples sizes and red indicating larger sample sizes.

Pooling *p*-values across all disciplines, there was strong evidence for “evidential value”; that is, researchers appear to be predominantly studying phenomena with nonzero effect sizes, as shown by the strong right skew of the p-curve for *p*-values found in both the Results (binomial glm: estimated proportion of *p*-values in the upper bin (0.025 ≤ *p* < 0.05) (lower CI, upper CI) = 0.257 (0.254, 0.259), *p* < 0.001, n = 14 disciplines) and the Abstracts (binomial glm: estimated proportion of *p*-values in the upper bin (0.025 ≤ p < 0.05) (lower CI, upper CI) = 0.262 (0.257, 0.267), *p* < 0.001, n = 10 disciplines). We found significant evidential value in every discipline represented in our text-mining data, irrespective of whether we tested the *p*-values from the Results or Abstracts (Table 1; Table 2). Based on the net trend across all disciplines, however, there was also strong evidence for p-hacking in both the Results (binomial glm: estimated proportion of *p*-values in the upper bin (0.045 < *p* < 0.05) (lower CI) = 0.546 (0.536), *p* < 0.001, n = 14 disciplines) and the Abstracts (binomial glm: estimated proportion of p-values in the upper bin (0.045 < p < 0.05) (lower CI) = 0.537 (0.518), *p* < 0.001, n = 10 disciplines). In most disciplines, there were more *p*-values in the upper than the lower bin; and when we look at the *p*-values text-mined from Results sections in every discipline where we had good statistical power (i.e., Health and medical Sciences, Biological Sciences, and Multidisciplinary), this difference was statistically significant (Table 1, Fig. 3A). When looking at *p*-values text-mined from Abstracts, despite the significant general trend, only the multidisciplinary and Information and Computer Science categories were significant (Table 2, Fig. 3B).

**Table 1:** Tests for evidential value and p-hacking across disciplines, using *p*-values obtained from the Results section.

Discipline	Number of *p*-values between 0 and 0.025	Number of *p*-values between 0.025 and 0.05	Binomial test for evidential value	Number of *p*-values between 0.04 and 0.045	Number of *p*-values between 0.045 and 0.05	Binomial test forp-hacking
Agricultural and veterinary sciences	375	125	<0.001	10	16	0.163
Biological sciences	11,074	3,562	<0.001	350	423	0.005
Chemical sciences	380	110	<0.001	14	17	0.360
Earth sciences	76	25	<0.001	0	4	0.063
Education	280	101	<0.001	9	8	0.685
Engineering	471	183	<0.001	16	12	0.828
Environmental sciences	657	190	<0.001	10	19	0.068
Information and computing sciences	790	266	<0.001	20	30	0.101
Mathematical sciences	72	22	<0.001	3	0	1.000
Medical and health sciences	45,460	16,537	<0.001	1,477	1,785	<0.001
Multidisciplinary	21,209	6,793	<0.001	638	750	0.001
Psychology and cognitive sciences	1,355	487	<0.001	29	50	0.012
Studies in human society	139	45	<0.001	8	3	0.967
Technology	94	37	<0.001	3	3	0.656

Number of *p*-values in each bin is the mean number based on 1,000 bootstraps of one *p*-value per Results section, rounded to the nearest whole number. Disciplines (n = 8) for which we found fewer than 50 *p*-values below 0.05 in the Results section were excluded.

**Table 2:** Tests for evidential value and p-hacking across disciplines, using *p*-values obtained from the Abstract.

Discipline	Number of *p*-values between 0 and 0.025	Number of *p*-values between 0.025 and 0.05	Binomial test for evidential value	Number of *p*-values between 0.04 and 0.045	Number of *p*-values between 0.045 and 0.05	Binomial test for p-hacking
Agricultural and veterinary sciences	96	35	<0.001	3	2	0.813
Biological sciences	1,787	632	<0.001	54	66	0.158
Chemical sciences	76	31	<0.001	3	4	0.500
Education	88	22	<0.001	2	0	1.000
Engineering	121	52	<0.001	2	1	0.875
Environmental sciences	42	15	<0.001	2	2	0.688
Information and computing sciences	251	105	<0.001	5	15	0.021
Medical and health sciences	18,428	6,692	<0.001	633	692	0.056
Multidisciplinary	5,056	1,621	<0.001	123	174	0.002
Psychology and cognitive sciences	98	37	<0.001	1	5	0.109

Number of *p*-values in each bin is the mean number based on 1,000 bootstraps of one *p*-value per Abstract, rounded to the nearest whole number. Disciplines (n = 12) for which we found fewer than 50 *p*-values below 0.05 in the Abstract were excluded.

Our text-mining suggests that p-hacking is widespread. Other studies that have inspected p-curves for far smaller sets of journals have also found evidence of p-hacking [12,40,45]. By contrast, Jager and Leek [3] found no evidence of p-hacking in a text-mining study of five medical journals. However, they were criticized for using *p*-values from Abstracts [46], because reporting *p*-values in Abstracts is optional, so they are more likely to contain only the strongest results (i.e., smallest *p*-values). Such a bias would exaggerate evidential value in our analysis, and make it harder to detect p-hacking (e.g., if researchers censor results with *p* = 0.049 from the Abstract, but not *p* = 0.041). Even though Abstracts are more likely to contain *p*-values that relate to primary hypotheses, which are expected to be more strongly p-hacked than *p*-values from less interesting, ancillary tests [41], lower power and reporting bias may impede detection of p-hacking using *p*-values obtained from Abstracts. The fact that we find evidence for p-hacking when using *p*-values from either the Abstracts or the Results sections across all scientific disciplines for which data are available (our overall analysis) supports the conclusion that p-hacking is rife.

Although we present evidence that p-hacking is widespread, there was still a strong right skew in all the p-curves we examined. This is consistent with researchers investigating predictions that lead to refutation of the null hypothesis, implying that the average true effect size studied by life scientists is nonzero. Given recent concerns about the lack of reproducibility of findings (e.g., [49] but see [50]) and the possibility that many published results are false [2], our results are reassuring. It is, of course, important to note that when using text-mining, we are combining many different types of questions to generate our p-curves. Consequently, it remains unclear whether there are some research fields or questions subsumed within the disciplines we considered for which the average effect size of published results is zero (i.e., the p-curve is flat). To examine this, it is important to also look at p-curves for well-defined research questions [41].

## The Consequences of P-Hacking for Meta-analyses

Meta-analysis is an excellent method for systematically synthesizing the literature and quantifying an effect or relationship by averaging effect sizes from multiple studies after weighting each one by its reliability [33,51]. However, meta-analyses are only as good as the data they use, and a recent study estimated that up to 37% of meta-analyses of clinical trials reporting a significant mean effect size represent false positives [34].

Tests for evidential value and p-hacking can readily be used to detect biases in datasets used in meta-analyses. We encourage researchers conducting meta-analyses to report *p*-values associated with each effect size (which is not currently standard practice) and then to test for evidential value and p-hacking. For a recent example of this practice, see [52]. To demonstrate this procedure, we obtained *p*-values from studies subject to meta-analyses by evolutionary biologists studying sexual selection [53–61] (see S1 Text).

When conducting our own meta-analysis of all the data used in these meta-analyses, there was clear evidence that researchers have strong evidential value for claims that effect sizes are nonzero (binomial glm: estimated proportion of *p*-values in the upper bin (0.025 ≤ p < 0.05) (lower CI, upper CI) = 0.202 (0.179, 0.228), *p*<0.001, n = 12 datasets). We then examined each dataset separately and found statistically significant evidential value for 9 of the 12 p-curves (Table 3). The three p-curves that did not show evidential value had the three lowest sample sizes, so low statistical power to detect evidential value may explain the lack of significance. Again, it is worth noting that evidential value for well-studied phenomena is not a given (see a real-world example in [62]).

**Table 3:** Tests for evidential value and p-hacking for published meta-analyses.

Meta-analysis	Number of *p*-values between 0 and 0.025	Number of *p*-values between 0.025 and 0.05	Binomial test for evidential value	Number of *p*-values between 0.04 and 0.045	Number of *p*-values between 0.045 and 0.05	Binomial test for p-hacking
Ackay & Roughgraden 2007 (1)	26	9	0.006	0 (0)	1 (1)	0.500 (0.500)
Cleasby & Nakagawa 2012	12	4	0.077	0 (0)	2 (1)	0.250 (0.500)
de Jong et al. 2012	22	1	<0.001	0 (0)	0 (0)	NA
Jiang et a.l 2013	318	82	<0.001	7 (7)	17 (11)	0.032 (0.240)
Kelly 2008 (1)	83	23	<0.001	3 (3)	5 (3)	0.363 (0.656)
Kelly 2008 (2)	91	18	<0.001	2 (2)	4 (2)	0.344 (0.688)
Kelly 2008 (3)	72	18	<0.001	3 (3)	6 (1)	0.254 (0.938)
Kraaijeveld et al. 2011	10	4	0.180	0 (0)	1 (1)	0.500 (0.500)
Prokop et al. 2012	79	15	<0.001	2 (2)	2 (2)	0.688 (0.688)
Santos et al. 2011	40	23	0.043	8 (8)	2 (2)	0.989 (0.989)
Weir et al. 2011 (1)	15	2	0.002	0 (0)	0 (0)	NA
Weir et al. 2011 (4)	9	2	0.065	0 (0)	0 (0)	NA

Meta-analyses with ten or fewer significant *p*-values are not shown. Numbers in the lower and upper bins of the p-hacking test are those including misreported *p*-values followed by those excluding misreported *p*-values in brackets.

**Fig 4:**
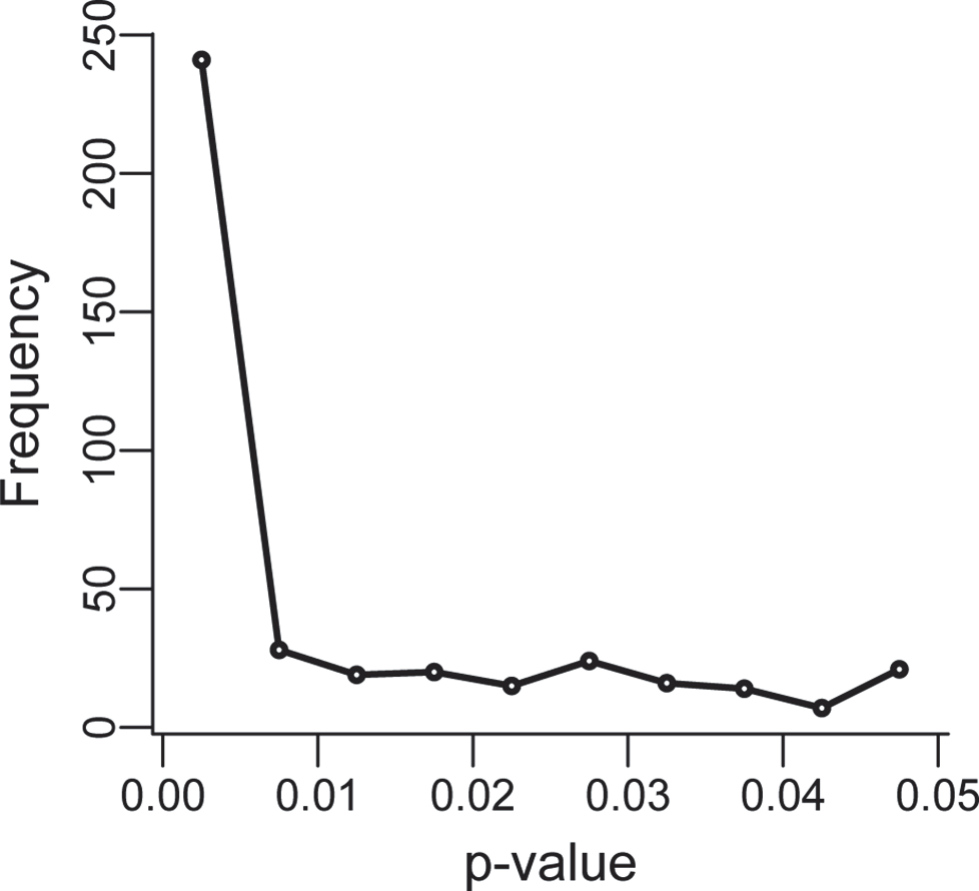
The distribution of *p*-values associated with the meta-analysis conducted by Jiang et al. (2013). The p-curve shows evidence for evidential value (strong right skew) and p-hacking (rise in *p*-values just below 0.05).

When considering evidence for p-hacking, we found that when we included misreported *p*-values (those given as *p* < 0.05 which were actually larger; a total of 16 cases—see S1 Text) there were more *p*-values in the upper than the lower bin for 7 of 12 p-curves (Table 3). This bias was significant in one dataset (Fig. 4), which was also the one with the largest sample size. However, the evidence for p-hacking disappeared when we excluded misreported *p*-values from our analyses (Table 3). One could argue that including misreported *p*-values in the upper bin of our binomial test biases our results toward detecting p-hacking, but reporting nonsignificant results as “*p*<0.05” is a component of p-hacking that should not be ignored. Indeed, Leggett et al. [45] also found considerable misreporting of *p*-values around the 0.05 threshold. They noted that *p*-values were more likely to be misreported as significant when they were not, rather than the reverse, and that this “error” has become more common in recent years.

More importantly, when misreported *p*-values were included in our analysis we found significant p-hacking from a meta-analysis of the p-curves of the 12 meta-analyses (binomial glm: estimated proportion of p-values in the upper bin (0.045 < *p* < 0.05) (lower CI) = 0.615 (0.513), p = 0.033; excluding misreported *p*-values: 0.489 (0.375), *p* = 0.443). Although questions subjected to meta-analysis might not be a representative sample of all research questions asked by scientists, our results indicate that studies on questions identified by researchers as important enough to warrant a meta-analysis tend to be p-hacked. Whether this influences the general conclusions of a meta-analysis depends on both the extent of p-hacking and the strength of the true effect. For instance, we found a statistically significant indication of p-hacking in only one of the 12 questions examined in published meta-analyses (Fig. 4). However, this study [56] also showed strong evidential value and *p*-values in the 0.045–0.05 bin were only a small proportion of published significant *p*-values. It is therefore unlikely that p-hacking would change the qualitative conclusions made in this meta-analysis, although p-hacking might have inflated the estimated mean effect size. In general, meta-analyses might be robust to inflated effects sizes that results from p-hacking, because: 1) all else being equal, studies that are most susceptible to p-hacking are those with small sample sizes (i.e., because low statistical power means less chance of a significant result), and these are given less weighting in a meta-analysis, 2) at least in some fields (e.g., ecology and evolution), meta-analyses often use data that is not directly related to the primary focus of the original paper. The *p*-values associated with secondary questions are less likely to be p-hacked. One way to check how sensitive estimates of effects sizes are to p-hacking would be to randomly remove the appropriate number of studies that contribute to a hump in the p-curve just below 0.05. Alternatively, meta-analysts could estimate effect sizes using p-curves (i.e., using only the significant *p*-values they find), a method which has been proposed to account for publication biases and to offer a conservative estimate of the true effect when there is p-hacking [62,63]. Development of p-curve methods is ongoing and we look forward to further tests of their ability to correct for the file-drawer effect, p-hacking, and other forms of publication bias given that real world data are likely to violate some of the assumptions in the available simulations of their effectiveness.

## Summary and Conclusions

Our study provides two lines of empirical evidence that p-hacking is widespread in the scientific literature. Our text-mining approach is based on a very large dataset that consists of *p*-values from different disciplines and questions, while our meta-analysis approach consists of *p*-values concerning a few specific hypotheses. Both approaches yielded similar results: evidential value for claims that the mean effect sizes for key study questions are nonzero—the conclusions researchers are making based on significant study findings—but that estimated mean effect size has probably been inflated by p-hacking.

Eliminating p-hacking entirely is unlikely when career advancement is assessed by publication output, and publication decisions are affected by the *p*-value or other measures of statistical support for relationships. Even so, there are a number of steps that the research community and scientific publishers can take to decrease the occurrence of p-hacking (see Box 3).

**Box 3. Recommendations**

The key to decreasing p-hacking is better education of researchers. Many practices that lead to p-hacking are still deemed acceptable. John et al. [16] measured the prevalence of questionable research practices in psychology. They asked survey participants if they had ever engaged in a set of questionable research practices and, if so, whether they thought their actions were defensible on a scale of 0–2 (0 = no, 1 = possibly, 2 = yes). Over 50% of participants admitted to “failing to report all of a study’s dependent measures” and “deciding whether to collect more data after looking to see whether the results were significant,” and these practices received a mean defensibility rating greater than 1.5. This indicates that many researchers p-hack but do not appreciate the extent to which this is a form of scientific misconduct. Amazingly, some animal ethics boards even encourage or mandate the termination of research if a significant result is obtained during the study, which is a particularly egregious form of p-hacking (Anonymous reviewer, personal communication).

**What can researchers do?**

- Clearly label research as prespecified (i.e., designed to answer a specific question, where detail of methods and analyses can be fully reported prior to data collection) or exploratory (i.e., involves exploration of data that looks intriguing, where methods and analyses used are often post hoc [13]), so that readers can treat results with appropriate caution. Results from prespecified studies offer far more convincing evidence than those from exploratory research [2].
- Adhere to common analysis standards [2]; measuring only response variables that are known (or predicted) to be important; and using sufficient sample sizes.
- Perform data analysis blind wherever possible. This approach makes it difficult to p-hack for specific results.
- Place greater emphasis on the quality of research methods and data collection rather than the significance or novelty of the subsequent findings when reviewing or assessing research. Ideally, methods should be assessed independently of results [13,44].

**What can journals do?**

- Provide clear and detailed guidelines for the full reporting of data analyses and results. For instance, stating that it is necessary to report effect sizes whether small or large, to report all *p*-values to three decimal places [27,64], to report samples sizes, and, most importantly, to be explicit about the entire analysis process (not just the final tests used to generate reported *p*-values). This will reduce p-hacking and aid the collection of data for meta-analyses and text-mining studies.
- Encourage and/or provide platforms for method prespecification [13,65]. Although methods and results in publications do not always match their prespecified protocols [5,66], prespecification allows readers to assess the risk of p-hacking and adjust their confidence in the reported outcomes accordingly.
- Encourage and/or provide platforms for open access to raw data. While access to raw data does not prevent p-hacking, it does make researchers more accountable for marginal results and allows readers to reanalyze data to check the robustness of results.

## Supporting Information

S1 Text Details of how text-mined data and data from meta-analyses were collected and analysed. (DOCX)

## Abbreviations:

NHST: Null hypothesis significance testing

## References

[1] Barch DM, Yarkoni T (2013) Introduction to the special issue on reliability and replication in cognitive and affective neuroscience research. Cogn Affect Behav Neurosci 13: 687–689. 10.3758/s13415-013-0201-7 23922199

[2] Ioannidis JPA (2005) Why most published research findings are false. PLoS Med 2: e124 16060722 10.1371/journal.pmed.0020124 PMC1182327

[3] Jager LR, Leek JT (2014) An estimate of the science-wise false discovery rate and application to the top medical literature. Biostatistics 15: 1–12. 10.1093/biostatistics/kxt007 24068246

[4] Begg CB, Berlin JA (1988) Publication bias—a problem in interpreting medical data. J R Stat Soc Ser A Stat Soc 151: 419–463.

[5] Dwan K, Altman DG, Arnaiz JA, Bloom J, Chan A-W, et al (2008) Systematic review of the empirical evidence of study publication bias and outcome reporting bias. PLoS ONE 3: e3081 10.1371/journal.pone.0003081 18769481 PMC2518111

[6] Fanelli D (2012) Negative results are disappearing from most disciplines and countries. Scientometrics 90: 891–904.

[7] Rosenthal R (1979) The file drawer problem and tolerance for null results. Psychol Bull 86: 638–641.

[8] Song F, Eastwood AJ, Gilbody S, Duley L, Sutton AJ (2000) Publication and related biases. Health technology assessment (Winchester, England) 4: 1–115. 10932019

[9] Sterling TD (1959) Publication decisions and their possible effects on inferences drawn from tests of significance—or vice versa. J Am Stat Assoc 54: 30–34.

[10] Stern JM, Simes RJ (1997) Publication bias: Evidence of delayed publication in a cohort study of clinical research projects. Br Med J 315: 640–645. 9310565 10.1136/bmj.315.7109.640 PMC2127436

[11] Laurance WF, Useche DC, Laurance SG, Bradshaw CJA (2013) Predicting publication success for biologists. Bioscience 63: 817–823.

[12] Brodeur A, Le M, Sangnier M, Zylberberg Y (2012) Star Wars: The empirics strike back. Paris School of Economics Working Paper 2012. http://ssrn.com/abstract=2089580.

[13] Cumming G (2014) The new statistics: Why and how. Psychol Sci 25: 7–29. 10.1177/0956797613504966 24220629

[14] Simmons JP, Nelson LD, Simonsohn U (2011) False-positive psychology: Undisclosed flexibility in data collection and analysis allows presenting anything as significant. Psychol Sci 22: 1359–1366. 10.1177/0956797611417632 22006061

[15] Gadbury GL, Allison DB (2014) Inappropriate fiddling with statistical analyses to obtain a desirable p-value: Tests to detect its presence in published literature. PLoS ONE 7: e46363. 10.1371/journal.pone.0046363 PMC3466248 23056287

[16] John LK, Loewenstein G, Prelec D (2012) Measuring the prevalence of questionable research practices with incentives for truth telling. Psychol Sci 23: 524–532. 10.1177/0956797611430953 22508865

[17] Hutton JL, Williamson PR (2000) Bias in meta-analysis due to outcome variable selection within studies. J R Stat Soc Ser C Appl Stat 49: 359–370.

[18] Bastardi A, Uhlmann EL, Ross L (2011) Wishful thinking: Belief, desire, and the motivated evaluation of scientific evidence. Psychol Sci 22: 731–732. 10.1177/0956797611406447 21515736

[19] Nosek BA, Spies JR, Motyl M (2012) Scientific utopia: II. Restructuring incentives and practices to promote truth over publishability. Perspect Psychol Sci 7: 615–631. 26168121 10.1177/1745691612459058 PMC10540222

[20] Fisher RA (1925) Statistical methods for research workers London: Oliver & Boyd.

[21] Benjamini Y, Hechtlinger Y (2014) Discussion: An estimate of the science-wise false discovery rate and applications to top medical journals by Jager and Leek. Biostatistics 15: 13–16. 10.1093/biostatistics/kxt032 24068247

[22] Goodman SN (1999) Toward evidence-based medical statistics. 1: The P value fallacy. Ann Intern Med 130: 995–1004. 10383371 10.7326/0003-4819-130-12-199906150-00008

[23] Sterne JAC, Smith GD (2001) Sifting the evidence—what's wrong with significance tests? Br Med J 322: 226–231. 11159626 10.1136/bmj.322.7280.226 PMC1119478

[24] Nickerson RS (2000) Null hypothesis significance testing: A review of an old and continuing controversy. Psychol Methods 5: 241–301. 10937333 10.1037/1082-989x.5.2.241

[25] Trafimow D (2003) Hypothesis testing and theory evaluation at the boundaries: Surprising insights from Bayes's theorem. Psychol Rev 110: 526–535. 12885113 10.1037/0033-295x.110.3.526

[26] Rohlf FJ, Sokal RR (1995) Statistical tables New York: W.H. Freeman.

[27] Ridley J, Kolm N, Freckleton RP, Gage MJG (2007) An unexpected influence of widely used significance thresholds on the distribution of reported P-values. J Evol Biol 20: 1082–1089. 17465918 10.1111/j.1420-9101.2006.01291.x

[28] Nakagawa S, Cuthill IC (2007) Effect size, confidence interval and statistical significance: A practical guide for biologists. Biol Rev Camb Philos Soc 82: 591–605. 17944619 10.1111/j.1469-185X.2007.00027.x

[29] Anderson DR, Burnham KP, Thompson WL (2000) Null hypothesis testing: Problems, prevalence, and an alternative. J Wildl Manage 64: 912–923.

[30] Loftus GR (1996) Psychology will be a much better science when we change the way we analyze data. Curr Dir Psychol Sci 5: 161–171.

[31] Benjamini Y, Yekutieli D (2005) False discovery rate-adjusted multiple confidence intervals for selected parameters J Am Stat Assoc 100: 71–81.

[32] Simonsohn U (2014c) Posterior-hacking: Selective reporting invalidates Bayesian results also. http://ssrncom/abstract=2374040.

[33] Koricheva J, Gurevitch J (2013) Place of meta-analysis among other methods of research synthesis In: Koricheva J, Gurevitch J, Mengersen K, editors. Handbook of met-analysis in ecology and evolution. Princeton, New Jersey: Princeton University Press.

[34] Pereira TV, Ioannidis JPA (2011) Statistically significant meta-analyses of clinical trials have modest credibility and inflated effects. J Clin Epidemiol 64: 1060–1069. 10.1016/j.jclinepi.2010.12.012 21454050

[35] Jennions MD, Moller AP, Hunt J (2004) Meta-analysis can "fail": Reply to Kotiaho and Tomkins. Oikos 104: 191–193.

[36] Kotiaho JS, Tomkins JL (2002) Meta-analysis, can it ever fail? Oikos 96: 551–553.

[37] Palmer AR (2000) Quasireplication and the contract of error: Lessons from sex ratios, heritabilities and fluctuating asymmetry. Annu Rev Ecol Sys 31: 441–480.

[38] Kelly CD (2006) Replicating empirical research in behavioural ecology: How and why it should be done but rarely ever is. The Quarterly Review of Biology 81: 221–236. 17051829 10.1086/506236

[39] Cumming G (2008) Replication and p intervals p values predict the future only vaguely, but confidence Intervals do much better. Perspectives on Psychological Science 3: 286–300. 26158948 10.1111/j.1745-6924.2008.00079.x

[40] Mariscampo EJ, Lalande DR (2012) A peculiar prevalence of p values just below .05. Q Rev Biol 65: 2271–2279. 10.1080/17470218.2012.711335 22853650

[41] Simonsohn U, Nelson LD, Simmons JP (2014a) P-curve: A key to the file drawer. J Exp Psychol Gen 143: 534–547. 10.1037/a0033242 23855496

[42] Wallis WA (1942) Compounding probabilities from independent significance tests. Econometrica 10: 229–248.

[43] Gerber AS, Malhotra N (2008) Publication bias in empirical sociological research—Do arbitrary significance levels distort published results? Sociol Methods Res 37: 3–30.

[44] Ho DE (2013) Foreword: Conference bias. J Empir Leg Stud 10: 603–611.

[45] Leggett NC, Thomas NA, Loetscher T, Nicholls MER (2013) The life of p: "Just significant" results are on the rise. Q J Exp Psychol 66: 2303–2309. 10.1080/17470218.2013.863371 24205936

[46] Ioannidis JPA (2014) Discussion: Why "An estimate of the science-wise false discovery rate and application to the top medical literature" is false. Biostatistics 15: 28–36. 10.1093/biostatistics/kxt036 24068251

[47] Clopper CJ, Pearson ES (1934) The use of confidence or fiducial limits illustrated in the case of the binomial. Biometrika 26: 404–413.

[48] Head ML, Holman L, Lanfear R, Kahn AT, Jennions MD (2015) Data from: The extent and consequences of p-hacking in science. Dryad Digital Repository. 10.5061/dryad.79d43. PMC4359000 25768323

[49] Nuzzo R (2014) Scientific method: Statistical errors. Nature 506: 150–152. 10.1038/506150a 24522584

[50] Klein RA, Ratliff KA, Vianello M, R.B. A, Bahník S, et al (2014) Data from investigating variation in replicability: A "many labs" replication project. J Open Psychol Data 2: e4.

[51] Sutton AJ, Abrams KR, Jones DR, Sheldon TA, Song F (2000) Methods for meta-analysis in medical research New york: John Wiley & Sons.

[52] Gildersleeve K, Haselton MG, Fales MR (2014) Meta-Analyses and p-curves support robust cycle shifts in women's mate preferencs: Reply to Wood & Carden (2014) and Harris, Pashler, and Mickes (2014). Psychol Bull 140: 1272–1280. 10.1037/a0037714 25180805

[53] Akcay E, Roughgarden J (2007) Extra-pair paternity in birds: Review of the genetic benefits. Evol Ecol Res 9: 855–868.

[54] Cleasby IR, Nakagawa S (2012) The influence of male age on within-pair and extra-pair paternity in passerines. Ibis 154: 318–324.

[55] de Jong K, Forsgren E, Sandvik H, Amundsen T (2012) Measuring mating competition correctly: available evidence supports operational sex ratio theory. Behav Ecol 23: 1170–1177.

[56] Jiang Y, Bolnick DI, Kirkpatrick M (2013) Assortative mating in animals. Am Nat 181: E125–E138. 10.1086/670160 23669548

[57] Kelly CD (2008) The interrelationships between resource-holding potential, resource-value and reproductive success in territorial males: How much variation can we explain? Behav Ecol Sociobiol 62: 855–871.

[58] Kraaijeveld K, Kraaijeveld-Smit FJL, Maan ME (2011) Sexual selection and speciation: The comparative evidence revisited. Biol Rev Camb Philos Soc 86: 367–377. 10.1111/j.1469-185X.2010.00150.x 20659104

[59] Prokop ZM, Michalczyk L, Drobniak SM, Herdegen M, Radwan J (2012) Meta-analysis suggests choosy females get sexy sons more than "good genes". Evolution 66: 2665–2673. 10.1111/j.1558-5646.2012.01654.x 22946794

[60] Santos ESA, Scheck D, Nakagawa S (2011) Dominance and plumage traits: Meta-analysis and metaregression analysis. Anim Behav 82: 3–19.

[61] Weir LK, Grant JWA, Hutchings JA (2011) The influence of operational sex ratio on the intensity of competition for mates. Am Nat 177: 167–176. 10.1086/657918 21460553

[62] Simonsohn U, Nelson LD, Simmons JP (2014b) P-Curve and effect size: Correcting for publication bias using only significant results. http://ssrncom/abstract=2377290 10.1177/1745691614553988 26186117

[63] van Assen MALM, van Aert RCM, Wicherts JM (2014) Meta-analysis using effect size distributions of only statistically significant studies Psychol Methods Advance Online Publication. 10.1037/met0000025 25401773

[64] Altman DG, Gore SM, Gardner MJ, Pocock SJ (1983) Statistical guidelines for contributors to medical journals. Br Med J 286: 1489–1493. 6405856 10.1136/bmj.286.6376.1489 PMC1547706

[65] Wagenmakers E-J (2007) A practical solution to the pervasive problems of p values. Psychon Bull Rev 14: 779–804. 18087943 10.3758/bf03194105

[66] Hahn S, Williamson PR, Hutton JL (2002) Investigation of within-study selective reporting in clinical research: follow-up of applications submitted to a local research ethics committee. J Eval Clin Pract 8: 353–359. 12164983 10.1046/j.1365-2753.2002.00314.x

