# Supplementary material for: The Extent and Consequences of P-Hacking in Science
Source: PLoS Biol. 2015 Mar 13;13(3):e1002106. doi: 10.1371/journal.pbio.1002106 (PMC4359000; doi:10.1371/journal.pbio.1002106)
Supplement: S1 Text — (DOCX) [file pbio.1002106.s001.docx]

**Supporting information**

**S1 Text**

*Obtaining p-values with text-mining*

We used text mining to search all open access papers available in the PubMed database. To do this, we first downloaded the open access subset of PubMed papers, available from (<ftp://ftp.ncbi.nlm.nih.gov/pub/pmc/articles.A-B.tar.gz>; <ftp://ftp.ncbi.nlm.nih.gov/pub/pmc/articles.C-H.tar.gz>; <ftp://ftp.ncbi.nlm.nih.gov/pub/pmc/articles.I-N.tar.gz>; <ftp://ftp.ncbi.nlm.nih.gov/pub/pmc/articles.O-Z.tar.gz>), on the 11^th^ of June 2014. We then used a python script to identify the Results and Abstract sections, and extract the p-values and associated metadata from each paper (e.g. DOI, journal name). Each paper is stored as an XML file, and for each paper we used the BeautifulSoup library (http://www.crummy.com/software/BeautifulSoup/bs4/doc/) to programmatically identify the results and abstract sections, and parse the p-values from those sections’ text. We defined p values as numbers in the range 0-1 that were preceded by “p =”, “p <”, “p >”, “p ≤” or “p ≥” regardless of spacing or capitalization (for the precise regular expressions used, see [[1](#_ENREF_1)]). The Python script we used to achieve this is available from ZENODO [[1](#_ENREF_1)].

We were unable to extract p-values from tables, because formatting varies widely among papers. We checked our results by manually extracting p-values (excluding those in tables) from 100 papers, selected at random from the collection of all papers for which we had found at least one p value. This analysis suggested that our error rates were low: of the 744 p-values we extracted by hand, we found only 15 cases in which the script failed to extract a reported p-value (2%). Of these, seven were due to our script being unable to parse scientific notation, four were due to p-values being reported in ranges or lists (e.g. “p = 0.02 – 0.04”), three were due to typographical errors in the published manuscript (e.g. “p = <0.00006”), and one was due to a p-value of exactly 1.0 (unimportant for the current analysis, which focuses only on p-values <0.05). We also found two false positives, both of which involved our script recording p-values from papers that describe alpha values in terms of a p-value threshold (e.g. ‘Statistical significance was taken as P < 0.05.’).

We also assigned each p-value to a scientific discipline based on the top level “Field of Research (FoR)” code of the Australian and New Zealand Standard Research Classification system (2012 version; <http://www.arc.gov.au/era/era_2012/era_journal_list.htm>) of the journal in which it was published. This system comprises 22 research disciplines. A number of journals (915/2844 in the initial sample) were not classified with a FoR code, and these were manually assigned to the most appropriate FoR category. The researcher responsible was blind to the p-values contained in the journal, ensuring unbiased assignment of journals to disciplines.

In this study, we use a subset of the full dataset described above. Because we are interested in exact p-values, we only use p-values reported as ‘p = ‘. To ensure that the data contributing to our p-curve are independent (see [[2](#_ENREF_2),[3](#_ENREF_3)]), we randomly sampled one p-value per paper. To address the potential stochasticity of sampling on one p-value per section (results or abstract) of each paper, we repeated this random sampling 1000 times, and took the average number of p-values in each bin across the 1000 samples for our p-curve analyses. These averages were rounded to the nearest whole number and then used in the sign tests described in Box 2 of our manuscript. The bootstrap analysis was performed separately on the p-values from the Abstracts, and on those from the Results. The full text-mined dataset, as well as the code used to produce the subset of the data we used in this analysis, and to perform the analyses we describe here, are deposited in the Dryad depository (<http://datadryad.org/review?doi=doi:10.5061/dryad.79d43>)*.*

*Analysis of p-values obtained with text-mining*

To quantify ‘evidential value’ (i.e. if there is evidence that the true effect size is non-zero) and p-hacking we constructed p-curves from the p-values we obtained (see Box 2). We restricted our analysis to exact p-values (i.e. reported as “p = ” and not as, for example, “p < ”). While this restriction excludes many published p-values from our analysis, it is necessary because we cannot know into which bin p-values reported as inequalities should be placed. If anything, excluding p-values presented as inequalities makes our test for p-hacking more conservative, because we suspect that a p-value is more likely to be abbreviated to p < 0.05 if it is p = 0.049 rather than, say, p = 0.041 (see Box 2). We present separate tests of evidential value and p-hacking for p-values extracted from the Results section, and for p-values extracted from the Abstracts. Abstracts are more likely than the Results to contain p-values exclusively associated with the main result of the paper, but, on the downside, they probably contain a biased sample of p-values (e.g. authors may be less likely to put p values just under 0.05 in the Abstract, making it harder for us to detect p-hacking in this dataset [[4](#_ENREF_4)]). For both analyses, we randomly selected one p-value per paper to ensure the independence of data and assigned each p-value to a scientific discipline based on the journal from which it was obtained. To attempt to account for the stochasticity in the results based on this random sampling of p-values, we analysed the average results of 1000 independent random samples of p-values (see above).

Researchers have identified weaknesses in the use of text-mined data to look for publication bias. One concern is that p-values are reported with varying precision i.e. to two or three decimal places, using various thresholds (e.g. p<0.05), or ‘eyecatchers’ (e.g. asterisks) instead of numbers. This makes it difficult to obtain all published p-values using text-mining, and might lead to under- or over-representation of certain values. Here we alleviate these issues by extracting only p-values reported with an equals sign, and specifying upper and lower bin values in our statistical test for p-hacking that excluded p-values only reported to two decimal places. Even so, we still make some assumptions. For instance, we assume that values above and below our test threshold are equally likely to be rounded to two decimal places. A second concern is the over-representation of some papers or tests. For example, primary data that give p-values close to the significance threshold might be more likely to be re-analysed and reported multiple times to demonstrate the robustness of the finding. We dealt with this concern by only using one p-value per paper picked randomly from the Results/Abstract section. A third concern with text-mining is that it gathers not only the p-values associated with primary study questions, but also those from less interesting ancillary tests (e.g. tests that the two groups did not differ prior to treatment), and these ancillary tests are less likely to be p-hacked [[3](#_ENREF_3)]. This means our analysis (especially the one focusing on the Results section) might under-estimate the frequency of p-hacking for key results, which is worrying because we nevertheless still found strong evidence for p-hacking.

*Obtaining p-values associated with meta-analyses*

We obtained p-values associated with studies used in meta-analyses by evolutionary biologists studying sexual selection. We chose this field as our case study because it is familiar to us, maximizing the likelihood that we correctly selected and extracted the relevant p values from the primary data papers. Unfortunately, the data sets accompanying published meta-analysis do not include the precise p-values for each effect size. These must therefore be manually extracted from primary papers, which is a labour-intensive task. To identify suitable meta-analyses for data extraction we searched the ISI Web of Science using the search term “sexual selection” AND meta-ana* OR metaanal (search conducted November 2013). We restricted our search to meta-analyses published from 2005 onward, because information about the sources of data has improved in recent evolutionary biology meta-analyses. Our search returned 70 papers, of which we excluded 59 after reading the title or abstract; Studies were excluded if they: 1) were based solely on genetic correlations; 2) involved only a single species or genus; 3) appeared not to have calculated effect sizes; 4) were on humans; 5) had been subject to a more recent meta-analysis asking the same question; 7) used unconventional statistics.

For the remaining 21 meta-analyses we obtained the data (effect sizes and their source) used in the meta-analyses either from the paper itself and any associated online supplements, or by contacting the authors. At this stage, additional meta-analyses were excluded if: 1) they used fewer than 20 primary studies, since small data sets are unlikely to yield enough significant p-values to detect p-hacking, or 2) a large proportion of the data used to generate effect sizes did not have readily associated p-values. Effect sizes for which it is relatively easy to find appropriate p-values include those with associated test statistics (e.g. F, t, Χ^2^) or derived from actual p-values, whereas data that is relatively difficult to find appropriate p-values for includes means and measures of variation, which are often obtained from figures and have not been subject to formal hypothesis testing.

We were left with 9 meta-analyses, from which we could extract p-values to generate a p-curve [[5-13](#_ENREF_5)]. Three of these publications [[5](#_ENREF_5),[9](#_ENREF_9),[13](#_ENREF_13)] presented results for multiple biological questions, so we separated datasets for each question. This yielded 16 potential datasets for inclusion in our study. Here we only present p-curves for the 12 data sets that had more than 10 significant p-values, as it would be difficult to detect even extreme p-hacking with sample sizes smaller than this.

The ease of extracting p-values from the original papers varied considerably. For this reason we established a standardized protocol. First, we excluded any effect sizes in the meta-analysis where it was noted that the original paper had reported a non-significant p-value. Second, we downloaded all the original papers used to obtain the remaining effect sizes. Third, using the information recorded in the meta-analysis datasets (e.g. p-values, test statistics, means and sample sizes used to calculate effect sizes) we located the p-value associated with the effect size reported in the meta-analysis. We did this even if a p-value was reported in the meta-analysis to ensure that they were recorded exactly as the original author had reported them (e.g. to the same number of decimal places). Fourth, if the original p-value was reported as “less than” or only to two decimal places we recalculated p-values from data/test statistics in the original paper (matching the original paper’s analysis as closely as possible). If the recalculated p value differed from that in the original paper we used the 3 decimal value closest to that reported in the original paper. In 16 cases p-values reported as < 0.05 were actually calculated to be greater than 0.05 (range = 0.051 – 0.178, mean = 0.093). Previous studies of p-hacking have coded this kind of misreported p-value as significant (see [[14](#_ENREF_14)]). Here, we treat these values in two ways: (a) by coding these values as 0.049, and (b) by repeating analyses with these p-values excluded. Data deposited in the Dryad depository (<http://datadryad.org/review?doi=doi:10.5061/dryad.79d43>)*.*

**References**

1. Lanfear R (2014) pvalues version 0.1 doi:105281/zenodo.13147.

2. Gerber AS, Malhotra N (2008) Publication bias in empirical sociological research - Do arbitrary significance levels distort published results? Sociological Methods & Research 37: 3-30.

3. Simonsohn U, Nelson LD, Simmons JP (2014a) P-curve: A key to the file drawer. Journal of Experimental Psychology: General 143: 534-547.

4. Ioannidis JPA (2014) Discussion: Why "An estimate of the science-wise false discovery rate and application to the top medical literature" is false. Biostatistics 15: 28-36.

5. Akcay E, Roughgarden J (2007) Extra-pair paternity in birds: Review of the genetic benefits. Evolutionary Ecology Research 9: 855-868.

6. Cleasby IR, Nakagawa S (2012) The influence of male age on within-pair and extra-pair paternity in passerines. Ibis 154: 318-324.

7. de Jong K, Forsgren E, Sandvik H, Amundsen T (2012) Measuring mating competition correctly: available evidence supports operational sex ratio theory. Behavioral Ecology 23: 1170-1177.

8. Jiang Y, Bolnick DI, Kirkpatrick M (2013) Assortative mating in animals. American Naturalist 181: E125-E138.

9. Kelly CD (2008) The interrelationships between resource-holding potential, resource-value and reproductive success in territorial males: How much variation can we explain? Behavioral Ecology and Sociobiology 62: 855-871.

10. Kraaijeveld K, Kraaijeveld-Smit FJL, Maan ME (2011) Sexual selection and speciation: The comparative evidence revisited. Biological Reviews 86: 367-377.

11. Prokop ZM, Michalczyk L, Drobniak SM, Herdegen M, Radwan J (2012) Meta-analysis suggests choosy females get sexy sons more than "good genes". Evolution 66: 2665-2673.

12. Santos ESA, Scheck D, Nakagawa S (2011) Dominance and plumage traits: Meta-analysis and metaregression analysis. Animal Behaviour 82: 3-19.

13. Weir LK, Grant JWA, Hutchings JA (2011) The influence of operational sex ratio on the intensity of competition for mates. American Naturalist 177: 167-176.

14. Leggett NC, Thomas NA, Loetscher T, Nicholls MER (2013) The life of p: "Just significant" results are on the rise. Quarterly Journal of Experimental Psychology 66: 2303-2309.
